# Gastropod Seed Dispersal: An Invasive Slug Destroys Far More Seeds in Its Gut than Native Gastropods

**DOI:** 10.1371/journal.pone.0075243

**Published:** 2013-09-25

**Authors:** Tamara Blattmann, Steffen Boch, Manfred Türke, Eva Knop

**Affiliations:** 1 Institute of Ecology and Evolution, University of Bern, Bern, Switzerland; 2 Institute of Plant Sciences and Botanical Garden, University of Bern, Bern, Switzerland; 3 Department of Ecology and Ecosystem Management, Technische Universität München, Freising-Weihenstephan, Germany; Norwegian University of Science and Technology, Norway

## Abstract

Seed dispersal is one of the most important mechanisms shaping biodiversity, and animals are one of the key dispersal vectors. Animal seed dispersal can directly or indirectly be altered by invasive organisms through the establishment of new or the disruption of existing seed dispersal interactions. So far it is known for a few gastropod species that they ingest and defecate viable plant seeds and consequently act as seed dispersers, referred to as gastropodochory. In a multi-species experiment, consisting of five different plant species and four different gastropod species, we tested with a fully crossed design whether gastropodochory is a general mechanism across native gastropod species, and whether it is altered by the invasive alien slug species *Arion lusitanicus.* Specifically, we hypothesized that a) native gastropod species consume the seeds from all tested plant species in equal numbers (have no preference), b) the voracious invasive alien slug *A. lusitanicus* – similarly to its herbivore behaviour – consumes a higher amount of seeds than native gastropods, and that c) seed viability is equal among different gastropod species after gut passage. As expected all tested gastropod species consumed all tested plant species. Against our expectation there was a difference in the amount of consumed seeds, with the largest and native mollusk *Helix pomatia* consuming most seeds, followed by the invasive slug and the other gastropods. Seed damage and germination rates did not differ after gut passage through different native species, but seed damage was significantly higher after gut passage through the invasive slug *A. lusitanicus*, and their germination rates were significantly reduced.

## Introduction

Animals are important dispersal vectors of plant propagules [1] which is crucial for biodiversity, plant population dynamics, species distributions, and gene flow [2], [3], [4]. Beside various vertebrates and insects, also gastropods have been discovered as endozoochorous (transport of propagules inside animals) seed dispersal vectors of propagules [5], [6], [7], [8], [9], [10]. This phenomenon has been described as gastropodochory [11], but documented only for a limited number of gastropod species (mostly slug species) and plant species (see references above).

Seed dispersal mechanisms are currently threatened by several anthropogenic factors such as biological invasions [12]. Invasive alien species successfully establish and spread outside their native range, and they often cause enormous economic costs and ecological damage [13]. They might either directly or indirectly disrupt seed-dispersal interactions. For example, they directly influence the local seed-dispersal mechanism by consuming fruits which would be dispersed by native species [14]. Indirectly, they might influence the abundance or the behaviour of a native seed disperser [12]. Direct and indirect interaction changes might have a negative impact on the resident biodiversity. Evidence for a negative outcome is well known from invasive ants and vertebrates, which indirectly disrupted the seed dispersal by displacing native seed dispersers [15]. On the other hand, examples of direct disruptions are very scarce (but see [14], [16]). However, also positive out-comes may occur. For example, Hansen et al. [17] showed that an alien species replaced the dispersal service of a native species which became extinct.

Beside various vertebrates and insects, many gastropods are listed as being invasive species (www.invasive.org) worldwide [18]. In Europe, the slug *Arion lusitanicus* Mabille (synonym *A. vulgaris* Moquin-Tandon) is suggested to be the most invasive terrestrial gastropod [19]. Despite its invasiveness all over Europe, to our knowledge, there is nothing known on its impact on the native biodiversity and ecosystem functioning. Yet it is, however known as a very voracious species compared to other species [10].

The impact of endozoochorous plant dispersal on the plant species distribution and their population dynamics depends on the quality and quantity of the dispersed seeds, for example how the seeds survive gut passage [20]. Here, we tested with a fully crossed design whether gastropodochory is a general mechanism across native gastropod species, and whether it is altered by the invasive alien slug species *Arion lusitanicus.* Specifically, we hypothesized that a) native gastropod species consume the seeds from all tested plant species in equal numbers (independent from seed or fruit type), b) the voracious invasive alien slug *A. lusitanicus* – similarly to its herbivore behaviour – consumes a higher amount of seeds than native gastropods, and that c) seed viability is equal among different gastropod species after gut passage.

## Materials and Methods

In a multi-species approach, we fed four gastropod species with five plant species and assessed number of consumed seeds, damage rates after gut passage, and germination rates. No permissions were required to collect and work with the used specimens, and none of the specimens was a protected species.

### Study Species

We used four naturally co-occurring gastropod species including the two slug species *Arion lusitanicus* and *A. rufus* (L.), as well as the two snail species *Cepaea nemoralis* L. and *Helix pomatia* L. *Arion rufus* is closely related to *A. lusitanicus*. They share the same habitats and are as adults hard to distinguish by external characters. As juveniles, however, *A. rufus* has a light brownish-orange colour whilst *A. lusitanicus* is darker with black stripes [21]. *Arion lusitanicus* is listed as one of the 100 most invasive species in Europe [19] but its origin is currently unknown [22]. In Switzerland, however, first records of *A. lusitanicus* date back to the year 1950 [23]. Since then, this species has successfully invaded all lowland parts of Switzerland and is now very common in gardens and cultural land, where it widely replaced *A. rufus* [24]. For these reasons and as only small populations of *A. rufus* are left in the Swiss lowlands, we used individuals descended from specimens collected in the wild, which have been identified after egg deposition. Individuals of both slug species were of the same age (8–10 month). Adult snail individuals were collected in the wild and therefore the actual age is unknown. The parental specimens of the two slug species and the two snail species were collected in lowland forests of central Switzerland: *Arion lusitanicus, C. nemoralis, H. pomatia* (46°57′N, 7°25′E), *A. rufus* (47°21′N, 8°18′E), respectively. Furthermore, we selected five experimental plant species, which co-occur on fallow grounds and crop fields: *Agrostemma githago* L., *Brassica napus* L., *Camelina sativa* (L.) Crantz, *Melilotus albus* Medik., and *Valerianella locusta* (L.) Laterr. Up to now for no particular animal-mediated seed dispersal strategies are known [25]. It is, however, known that seed dispersal mechanisms are diverse and fundamental for the weed flora present in the agroecosystem [26] because arable weeds are composed mainly of therophyte species [27] whose annual character obliges them to rely exclusively on seed production and shatter for survival of the progenies [28]. The seeds used in the experiment were bought from Rieger-Hofmann GmbH (Blaufelden-Raboldshausen, D; *M. albus*, *A. githago*) and fenaco (UFA-Samen Winterthur, CH; *B. napus*, *C. sativa*, *V. locusta*).

### Experimental Setup

All experiments were conducted in a climate chamber under standardised conditions (d/n: 14/10 h, 18/16°C) between August 2010 and September 2011. We kept gastropod individuals separately in plastic arenas (19.0 cm × 13.5 cm × 6.0 cm), which contained wet paper towels to ensure humid conditions. We randomly formed three groups consisting of 25 individuals of each gastropod species. Within a group, every individual received 30 seeds of the same plant species. To ensure that the seeds of each plant species were offered to all gastropod species, two groups per gastropod species were therefore used twice. One experimental run consisted of three stages. First, the gastropods starved for two days. Secondly, 30 seeds of one plant species were exposed to each of the 25 individuals of one gastropod group for three days. Together with the seeds, a sterilised beech leaf was offered as alternative food to prevent that the gastropods were forced to feed on the seeds [5]. Thirdly, after seed consumption the gastropods were individually weighed and fed with vegetable food (carrot, potato, and zucchini) for another two days.

After the three days of seed exposure to the gastropods, the remaining seeds were collected and counted per individual. The faeces in the plastic arenas were checked for seeds and the number of seeds apparently damaged was counted. Seeds were categorised as damaged when only their fragments could be found in the faeces and future germination could be excluded. All faeces (including those with damaged seeds) were then put on wet soil and kept in a climate chamber (d/n: 14/10 h, 18/16°C) to evaluate the number of germinating seeds per individual in relation to the number of previously consumed seeds. The gastropod arenas were controlled daily for further faeces containing seeds for one week after seed consumption to ensure the collection of seeds with longer gut retention times. Seeds with a delayed gut passage were also transferred to soil. We daily recorded germination rate until two months after the last germination of a particular plant species. Simultaneously, we also measured the germination rate of the experimental plant species without gut passage: 90 seeds of each plant species divided into three control groups were first put on wet paper towels for three days and afterwards transferred to wet soil to evaluate the germination rates. Until two months after the last germination event, the number of germinated seeds was counted.

### Statistical Approach

We analysed the number of consumed seeds, damage rates, and germination rates using generalized linear mixed effect models (GLMMs). For the analyses of the number of consumed seeds, we used plant species (five levels: *A. githago*, *B. napus*, *C. sativa*, *M. albus,* and *V. locusta*) as well as the gastropod species (four levels: *A.* *lusitanicus*, *A. rufus*, *C. nemoralis*, *H. pomatia*) as fixed factors. The weight (log-transformed) of the used specimens was used as a covariate and the individual specimens as random factors (e.g. some specimens were used for two plants, in total of 363 specimens per level and a total of 707 observations). A Poisson error-distribution was assumed. The quantiles of the standardized residuals of the full model were plotted against the quantiles of a normal distribution to assess the assumption of the residual distribution. A possible overdispersion was estimated by including an observation-level random factor (as many levels as observations) into the full model and comparing it to a model without this additional variance parameter [29]. When the observation-level random factor significantly improved the model, it was retained in the model for the subsequent model selection. The minimal adequate model was searched by stepwise deletion of non-significant predictors and interactions. Significances were assessed by comparing models with and without the factor under question using likelihood ratio tests. To describe sample uncertainty of the estimated mean for each species-plant combination we used Bayesian methods as recommended for mixed models [30]. The function sim from the R-package arm was used to simulate 2000 samples from the joint posterior distribution of the model parameters of the minimal adequate model. From these 2000 random samples of sets of model parameters, 2000 fitted values for each status-plant combination were obtained. The 2.5% and 97.5% quantiles of these 2000 values were used as lower and upper limit of the 95% credible intervals (as shown in the Figures). Posterior probability whether the pairwise difference between the mean of *A. lusitanicus* and each of the three native gastropod species separately was bigger than zero was calculated for each plant species. In the same way, we also calculated whether the means of all native gastropod species together compared to *A. lusitanicus* is bigger than zero, for the plant species separately as well as pooled. In addition, we inspected the contrasts between the mean levels of the species, i.e., we inspected whether the species consumed different amounts of seeds after correcting for the effect of their weight and plant species. The estimated contrasts were computed using the glht function in the R-package multcomp [31]. P-values of multiple comparisons were corrected by the Holm correction. The damage rates were analysed using the same factors and statistical procedure as described for the consumption rate, except for the dependent variables, which were in this case the percentage of damaged seeds, and a Binomial error-distribution was assumed instead, and no contrasts were calculated. For analysis of the germination rate, the same factors and statistical procedures as described in the damage rate were applied except for the fixed factor gastropod species which was replaced by a treatment factor that also included a factor level for the control seeds without gut passage (five levels: control, *A. lusitanicus*, *A. rufus*, *C. nemoralis*, *H.* *pomatia*), and a Binomial error-distribution was assumed. All statistical analyses with R 2.12.1 [32], and GLMMs were calculated using the function glmer.

## Results

The mean (se) weight after seed consumption was 4.8 g (0.1) for *A. lusitanicus*, 2.8 g (0.1) for *A. rufus*, 3.6 g (0.1) for *C. nemoralis*, and 22.4 g (0.1) for *H. pomatia*.

**Figure 1:**
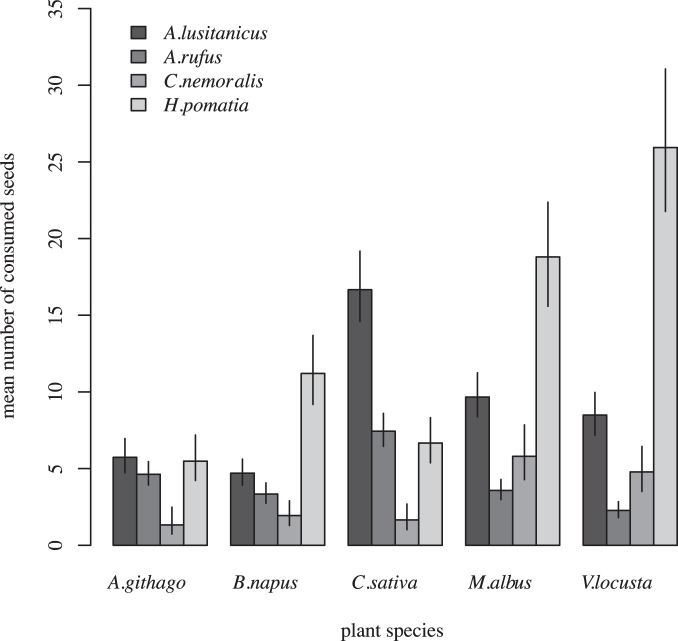
Fitted mean values and 95% credible interval of number of consumed seeds out of a total of 30 seeds per plant species. *A. lusitanicus:* N* = *250, *A. rufus:* N* = *375, *C. nemoralis:* N* = *125, and *H. pomatia:* N* = *125.

Number of consumed seeds: All predictors remained in the minimal adequate model: There was a significant effect of gastropod species (p<0.001), plant species (p<0.001), gastropod species × plant species (p<0.001) and log(weight) (p<0.001). The mean number of consumed seeds was significantly higher for *A.* *lusitanicus* compared to the native gastropod species, except for *H.* *pomatia* which consumed significantly more seeds of three plant species than *A. lusitanicus* (Fig. 1, Table S1). The contrasts inspecting the number of consumed seeds after correcting for the effect of plant species and gastropod weight revealed similar results (*A. rufus* – *A. lusitanicus* estimate = −0.541, se = 0.076, z = −7.085, p<0.001; *C. nemoralis* – *A. lusitanicus*: estimate = −0.951, se = 0.111, z = −8.570, p<0.001, *H. pomatia* – *A. lusitanicus*: estimate = −0.173, se = 0.141, z = −1.225, p = 0.578). The pattern of posterior probabilities was weaker when all native gastropod species were pooled and contrasted to *A. lusitanicus*: The posterior probabilities for the hypothesis that the invasive slug consumed more or equal number of seeds than native gastropods were: *A. githago*: p = 0.002, *B. napus*: p = 0.913, *C. sativa*: p<0.001, *M. albus*: p = 0.411, *V. locusta*: p = 0.992. For all gastropod species as well as all plant species pooled the posterior probability for the hypothesis that the invasive slug consumed a more or equal number of seeds was p<0.001.

Damage rate: The mean (se) damage rate per gastropod species were for *A. lusitanicus* = 54% (4), *A. rufus = *7% (2), *C. nemoralis* = 7% (3), *H. pomatia* = 15% (3). The mean (se) damage rate per plant species were for *A. githago* = 11% (3), *B. napus* = 23% (5), *C. sativa* = 22% (4), *M. albus* = 44% (5), *V. locusta* = 19% (4). Due to a lack of destroyed seeds in several plant species – gastropod species combination (*A. githago – C. nemoralis*, *A. githago – H. pomatia, B. napus – A. rufus, C. sativa – C. nemoralis*, *C. sativa – H. pomatia*, *V. locusta – A.* *rufus*), the interaction plant species × gastropod species could not be fitted to the full model and thus was discarded. All other predictors remained in the minimal adequate model: There was a significant difference between gastropod species (p<0.001), plant species (p<0.001), and a significant effect of log (weight) (p = 0.026). Overall, in all pairwise comparisons, the damage rate of seeds was significantly higher (p<0.001) for *A. lusitanicus* than for native gastropod species. This was also true when native gastropod species were pooled, plant species were pooled respectively.

**Figure 2:**
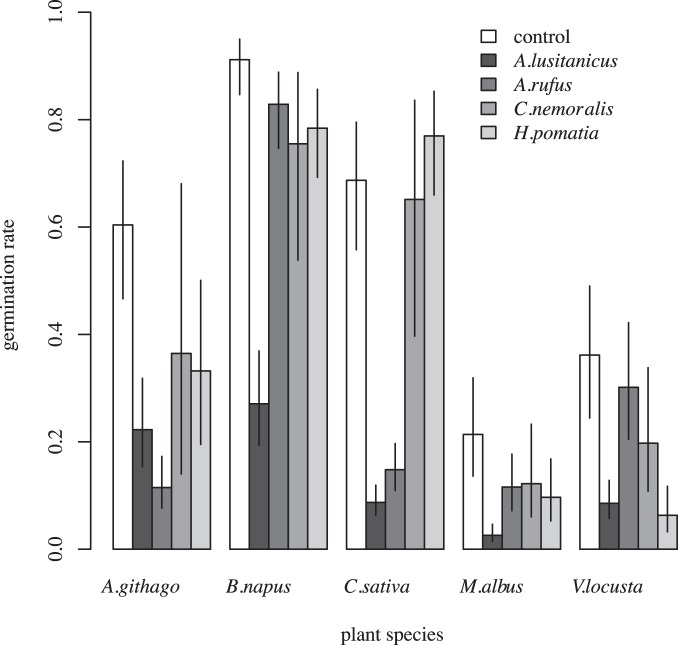
Fitted mean values and 95% credible interval of control seeds (white), seeds digested by the invasive species (dark grey), and the native gastropod species (light grey).

Germination rate: On average (se) the plant species required the following number of days until germination: *A. githago* = 6 (1), *B. napus* = 10 (1), *C. sativa* = 10 (1), *M. albus* = 21 (2), and *V. locusta* = 17 (1). All predictor variables remained in the minimal adequate model: There was a significant effect of treatment (p<0.001), plant species (p<0.001), and treatment × plant species (p<0.001). Germination rate was significantly lower after gut passage through *A. lusitanicus* than after gut passage through native gastropod species except for seeds of *A. githago* (Fig. 2, Table S2). This was also true when the pooled mean values of all native gastropod species were compared to *A. lusitanicus*, for plant species separately (*A. githago*: p = 0.234, *B. napus*: p<0.001, *C. sativa*: p<0.001, *M. albus*: p<0.001, *V. locusta*: p = 0.002) as well as pooled (p<0.001). Compared to control seeds, the germination rate was reduced in all plant species after gut passage through *A. lusitanicus* whereas no clear pattern was found for the native ones (Fig. 2, Table S3).

## Discussion

As expected the gastropod species differed in the amount of seeds consumed. Against our hypothesis, however, it was not the invasive *A. lusitanicus* consuming most seeds but the largest native mollusc *H. pomatia*, followed by the invasive slug and the other native gastropod species. Also after correcting for the gastropods weight and plant species effect, *H. pomatia* consumed significantly more seeds than the other species. This is surprisingly since Honek et al. [10] found a higher consumption rate of seeds and seedlings of *Taraxacum sp.* by *A. lusitanicus* compared to other gastropods including *H. pomatia*. However, they only investigated the seed consumption rate of one plant species. Also in our study, *A. lusitanicus* consumed significantly more seeds of single plant species (*C. sativa*) which stresses the need of multi-species studies for general conclusions in invasion ecology (e.g. [33]). Thus, on a more general level, when comparing the average number of consumed seeds by a native gastropod independent of its identity (i.e. the mean consumption rate of the investigated native gastropods after accounting for weight differences between them) with the average number of consumed seeds by *A. lusitanicus* showed, that the invasive species consumed on average a higher number of seeds. This is in line with the idea that a high up-take rate is a general trait of invasive species [34].

Contrary to most previous publications on gastropodochory [5], [7], [9], [10] in our study seeds had been partially destroyed in the feeding process or during digestion, and this happened significantly more often in *A. lusitanicus*. Previous studies, however, rarely used *A. lusitanicus* as a model organism and this is probably the reason why it has not been discovered so far (but see [10]). Differences in radula morphology or in the digestive tract could cause this higher number of destroyed seeds by *A. lusitanicus* but evidence is lacking so far. In general, a superior ability to exploit resources has previously been found to be a key mechanism explaining the success of invaders [35]. In addition to a higher up-take rate, a more efficient resource conversion of invasive species might explain their competitive superiority over native species (i.e. superior exploitative competition; [35]). The higher damage rate of the seeds consumed by *A. lusitanicus* compared to native gastropods suggests that *A. lusitanicus* more often digested the very nutrient rich seeds and thus might be more efficient in converting resources. This is in line with previous findings which show that *A. lusitanicus* is able to reproduce under low quality food conditions, suggesting that this species is either very efficient in converting resources or requires less nutrients than other slug species [36].

The reduced germination rate after seed passage through gastropod guts was surprising. In previous studies using gastropod species native to the respective regions, slug defecated seeds germinated as well as control seeds [5] or there was even a generally positive effect of slug gut passage on germination rate [7]. In the study of Gervais et al. [9], germination of *Disporum smithii* (Hook.) Piper was slightly enhanced but significantly less seeds germinated after gut passage through banana slugs (*Ariolimax columbianus* (Gould)) in *Rubus spectabilis* Pursh. In accordance to our study, Honek et al. [10] found that germination rate of dandelion seeds was reduced by 20% if ingested and defecated by *A. lusitanicus*. Nevertheless, germination rate was much higher than in our study. The significantly reduced germination rate after gut passage through *A. lusitanicus* suggests that this species could potentially disrupt the mutualistic plant-animal interaction directly by predating on the seeds rather than dispersing them, thereby constituting one of the rare examples of how an invader can directly disrupt the local seed dispersal process (but see [14], [16]). So far, most evidence comes from an indirect disruption, namely the replacement of native seed dispersers by an invasive species that does not disperse the seeds (e.g. [15], [17], [37], [38]). Even though seed dispersal disruptions are increasingly being reported from different ecosystems, few studies yet provide empirical evidence of the long-term costs of disruptions [39]. Traveset et al. [39], however, showed that it can lead to species regression and in the long term, even to local extinctions. As shown by Türke et al. [40], gastropods appear to substitute ants as seed dispersers in Central European beech forests. Interestingly, these forests are still inhabited by the native large slugs *A. rufus* and *A. ater* (L.) whereas *A. lusitanicus* is still absent (personal observation by M.Türke). Our results suggest that the invasion of these forests by *A. lusitanicus* –which already can be found in fragmented forests [41] – could disrupt this mutualistic relationship. In fact, the invasive slug *Arion fasciatus* (Nilsson) from Europe appeared to prevent dispersal of seeds by the common animal dispersers of blueberry, *Vaccinium angustifolium* Aiton, a vertebrate dispersed plant, and of *Asarum canadense* L., an ant-dispersed herb [42], in Canada. Therefore, as a next step, the long-term costs of the found mechanism have to be tested in the field. In summary, all investigated gastropods potentially disperse seeds of all investigated plant species, but the invasive slug *A. lusitanicus* destroyed significantly more seeds during gut passage than native gastropods, which leads to a significantly reduced germination rates. As the invasive species is dominating many habitats and is still spreading in Central Europe it is likely that the invasive species disrupts the mutualistic gastropod-plant interaction. Long-term field experiments, however, are needed to confirm this assumption.

## Supporting Information

Table S1 **Posterior probabilities calculated from 2000 simulated samples for the hypothesis that** ***A. lusitanicus*** **consumed less or equal number of seeds compared to native gastropod species (i.e.** ***A. lusitanicus*** **consumed compared to** ***A. rufus*** **with a probability of 0.008 less or equal number of seeds of** ***B. napus*** **meaning that it consumed significantly more seeds).**
(DOCX)

Table S2 **Probabilities calculated from 2000 simulated samples for the hypothesis that seeds germinate more or equally after gut passage through** ***A. lusitanicus*** **compared to native gastropod species (i.e. seeds of** ***B. napus*** **germinated with a probability of 0 more or equally after gut passage through** ***A. lusitanicus*** **compared to** ***A. rufus*** **).**
(DOCX)

Table S3 **Posterior probabilities calculated from 2000 simulated samples for the hypothesis that seeds germinate more or equally after gut passage through a gastropod species compared to control seeds (C).**
(DOCX)
