# Supplementary material for: Gastropod Seed Dispersal: An Invasive Slug Destroys Far More Seeds in Its Gut than Native Gastropods
Source: PLoS One. 2013 Sep 25;8(9):e75243. doi: 10.1371/journal.pone.0075243 (PMC3783466; doi:10.1371/journal.pone.0075243)
Supplement: Table S1 — Posterior probabilities calculated from 2000 simulated samples for the hypothesis that A. lusitanicus consumed less or equal number of seeds compared to native gastropod species (i.e. A. lusitanicus consumed compared to A. rufus with a probability of 0.008 less or equal number of seeds of B. napus meaning that it consumed significantly more seeds). (DOCX) [file pone.0075243.s001.docx]

|  | *A. lusitanicus- A. rufus* | *A. lusitanicus-C. nemoralis* | *A. lusitanicus-H. pomatia* |
| --- | --- | --- | --- |
| *A. githago* | 0.051 | < 0.001 | 0.403 |
| *B. napus* | 0.008 | < 0.001 | 1 |
| *C. sativa* | < 0.001 | < 0.001 | < 0.001 |
| *M. albus* | < 0.001 | 0.005 | 1 |
| *V. locusta* | < 0.001 | 0.002 | 1 |
