# Supplementary material for: Gastropod Seed Dispersal: An Invasive Slug Destroys Far More Seeds in Its Gut than Native Gastropods
Source: PLoS One. 2013 Sep 25;8(9):e75243. doi: 10.1371/journal.pone.0075243 (PMC3783466; doi:10.1371/journal.pone.0075243)
Supplement: Table S2 — Probabilities calculated from 2000 simulated samples for the hypothesis that seeds germinate more or equally after gut passage through A. lusitanicus compared to native gastropod species (i.e. seeds of B. napus germinated with a probability of 0 more or equally after gut passage through A. lusitanicus compared to A. rufus ). (DOCX) [file pone.0075243.s002.docx]

|  | *A. lusitanicus- A. rufus* | *A. lusitanicus-C. nemoralis* | *A. lusitanicus-H. pomatia* |
| --- | --- | --- | --- |
| *A. githago* | 0.992 | 0.172 | 0.121 |
| *B. napus* | < 0.001 | < 0.001 | < 0.001 |
| *C. sativa* | 0.01 | < 0.001 | < 0.001 |
| *M. albus* | < 0.001 | <0.001 | < 0.001 |
| *V. locusta* | < 0.001 | 0.016 | 0.792 |
