# Supplementary material for: Gastropod Seed Dispersal: An Invasive Slug Destroys Far More Seeds in Its Gut than Native Gastropods
Source: PLoS One. 2013 Sep 25;8(9):e75243. doi: 10.1371/journal.pone.0075243 (PMC3783466; doi:10.1371/journal.pone.0075243)
Supplement: Table S3 — Posterior probabilities calculated from 2000 simulated samples for the hypothesis that seeds germinate more or equally after gut passage through a gastropod species compared to control seeds (C). (DOCX) [file pone.0075243.s003.docx]

|  | *A. lusitanicus* - C | *A. rufus* - C | *C. nemoralis* - C | *H. pomatia* - C |
| --- | --- | --- | --- | --- |
| *A. githago* | <0.001 | <0.001 | 0.085 | 0.009 |
| *B. napus* | <0.001 | 0.032 | 0.018 | 0.004 |
| *C. sativa* | <0.001 | <0.001 | 0.340 | 0.854 |
| *M. albus* | <0.001 | 0.025 | 0.088 | 0.016 |
| *V. locusta* | <0.001 | 0.251 | 0.045 | <0.001 |
